# Supplementary material for: Exploring the Loci Responsible for Awn Development in Rice through Comparative Analysis of All AA Genome Species
Source: Plants (Basel). 2021 Apr 8;10(4):725. doi: 10.3390/plants10040725 (PMC8068336; doi:10.3390/plants10040725)
Supplement: Supplementary file 1 [file plants-10-00725-s001.zip › Supplementary_Figures.pptx]

## Slide 1
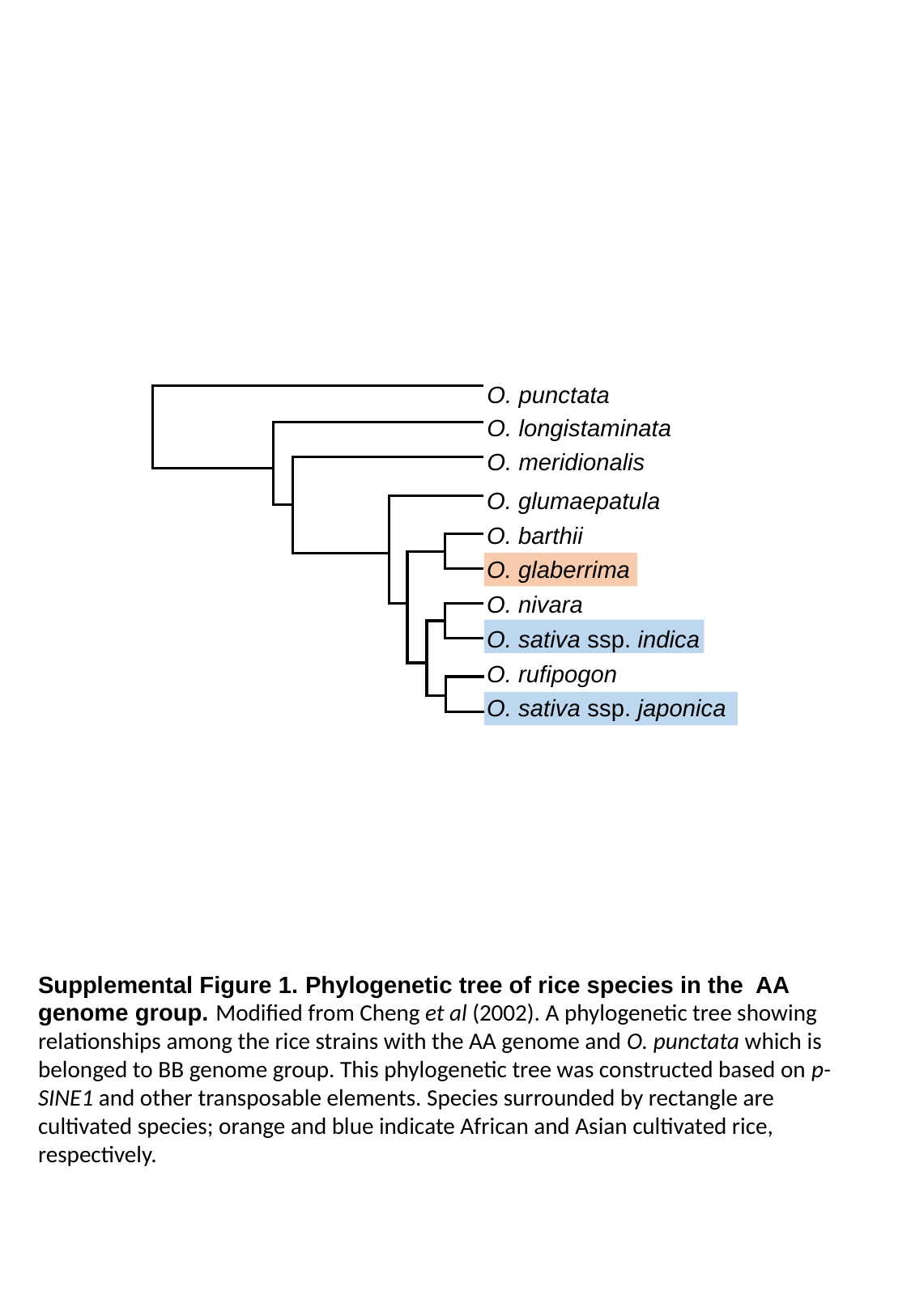

O. punctata
O. longistaminata
O. meridionalis
O. glumaepatula
O. barthii
O. glaberrima
O. nivara
O. sativa ssp. indica
O. rufipogon
O. sativa ssp. japonica
Supplemental Figure 1. Phylogenetic tree of rice species in the AA genome group. Modified from Cheng et al (2002). A phylogenetic tree showing relationships among the rice strains with the AA genome and O. punctata which is belonged to BB genome group. This phylogenetic tree was constructed based on p- SINE1 and other transposable elements. Species surrounded by rectangle are cultivated species; orange and blue indicate African and Asian cultivated rice, respectively.

## Slide 2
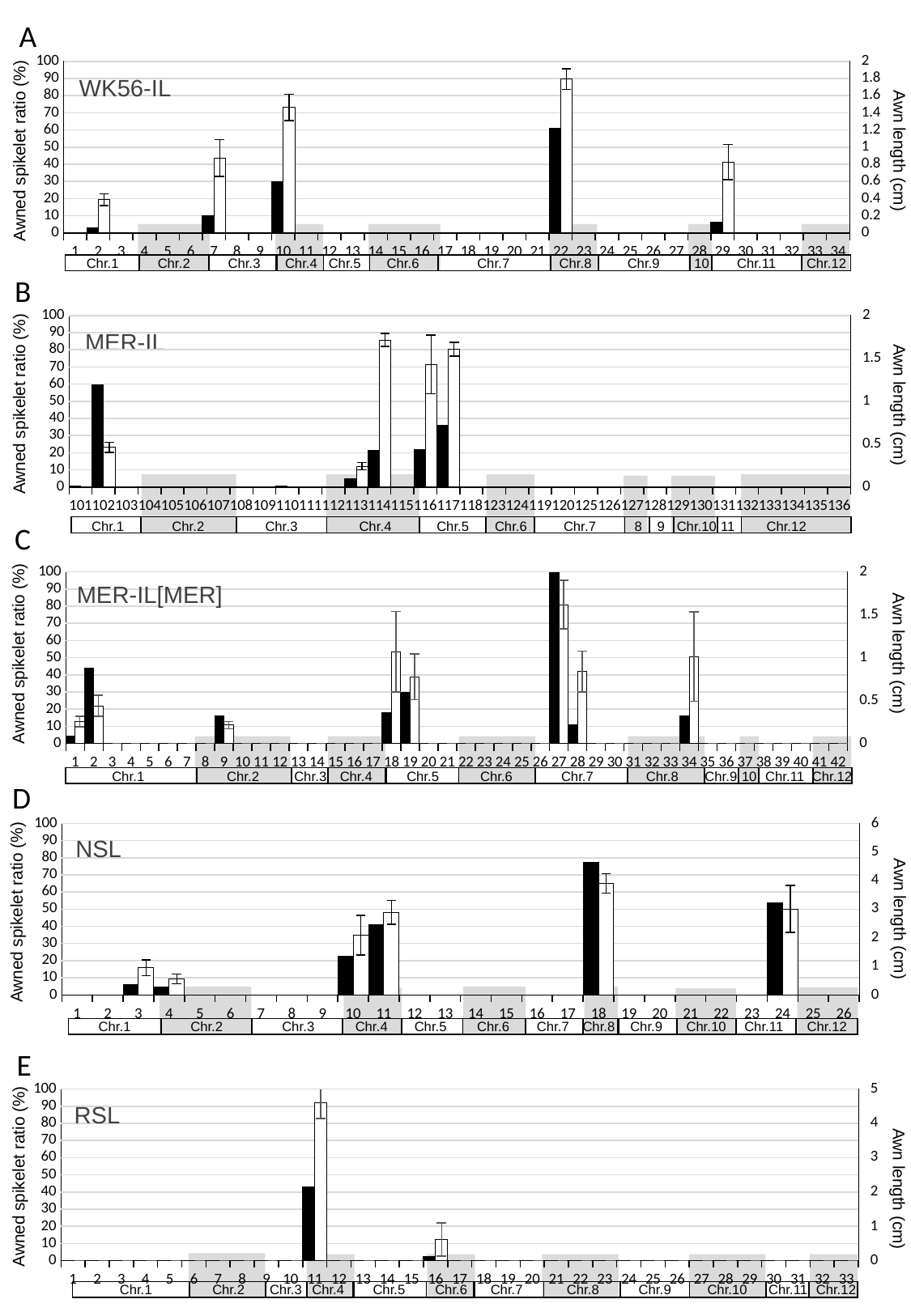

A
### Chart
| Category | awn ratio (%) | 1 | 2 | awn length (cm) |
|---|---|---|---|---|WK56-IL
Awned spikelet ratio (%)
Awn length (cm)
Chr.1
Chr.2
Chr.3
Chr.4
Chr.5
Chr.6
Chr.7
Chr.8
Chr.9
10
Chr.11
Chr.12
B
### Chart
| Category | awn ratio (%) | | | awn length(cm) |
|---|---|---|---|---|
| 101 | 0.8064516129032258 | None | None | 0.0 |
| 102 | 59.50246512746513 | None | None | 0.4636111111111111 |
| 103 | 0.0 | None | None | 0.0 |
| 104 | 0.0 | None | None | 0.0 |
| 105 | 0.0 | None | None | 0.0 |
| 106 | 0.0 | None | None | 0.0 |
| 107 | 0.0 | None | None | 0.0 |
| 108 | 0.0 | None | None | 0.0 |
| 109 | 0.0 | None | None | 0.0 |
| 110 | 0.7002098348690813 | None | None | 0.0 |
| 111 | 0.0 | None | None | 0.0 |
| 112 | 0.0 | None | None | 0.0 |
| 113 | 4.993081864391766 | None | None | 0.24341666666666667 |
| 114 | 21.422162491303173 | None | None | 1.7140625 |
| 115 | None | None | None | None |
| 116 | 21.9690218495935 | None | None | 1.4297569444444445 |
| 117 | 35.859214288626056 | None | None | 1.6063194444444444 |
| 118 | 0.0 | None | None | 0.0 |
| 123 | 0.0 | None | None | 0.0 |
| 124 | 0.0 | None | None | 0.0 |
| 119 | 0.0 | None | None | 0.0 |
| 120 | 0.0 | None | None | 0.0 |
| 125 | 0.0 | None | None | 0.0 |
| 126 | 0.0 | None | None | 0.0 |
| 127 | 0.0 | None | None | 0.0 |
| 128 | 0.0 | None | None | 0.0 |
| 129 | 0.0 | None | None | 0.0 |
| 130 | 0.0 | None | None | 0.0 |
| 131 | 0.0 | None | None | 0.0 |
| 132 | 0.0 | None | None | 0.0 |
| 133 | 0.0 | None | None | 0.0 |
| 134 | 0.0 | None | None | 0.0 |
| 135 | 0.0 | None | None | 0.0 |
| 136 | 0.0 | None | None | 0.0 |MER-IL
Awned spikelet ratio (%)
Awn length (cm)
Chr.1
Chr.2
Chr.3
Chr.4
Chr.5
Chr.6
Chr.7
8
9
Chr.10
11
Chr.12
C
### Chart
| Category | awn ratio (%) | 1 | 2 | awn length(cm) |
|---|---|---|---|---|MER-IL[MER]
Awned spikelet ratio (%)
Awn length (cm)
Chr.1
Chr.2
Chr.3
Chr.4
Chr.5
Chr.6
Chr.7
Chr.8
Chr.9
10
Chr.11
Chr.12
D
### Chart
| Category | awn ratio (%) | ダミー | ダミー２ | awn length (cm) |
|---|---|---|---|---|NSL
Awned spikelet ratio (%)
Awn length (cm)
Chr.1
Chr.2
Chr.3
Chr.4
Chr.5
Chr.6
Chr.7
Chr.8
Chr.9
Chr.10
Chr.11
Chr.12
E
### Chart
| Category | awn ratio (%) | ダミー | ダミー２ | awn length (cm) |
|---|---|---|---|---|RSL
Awned spikelet ratio (%)
Awn length (cm)
Chr.1
Chr.2
Chr.3
Chr.4
Chr.5
Chr.6
Chr.7
Chr.8
Chr.9
Chr.10
Chr.11
Chr.12

## Slide 3
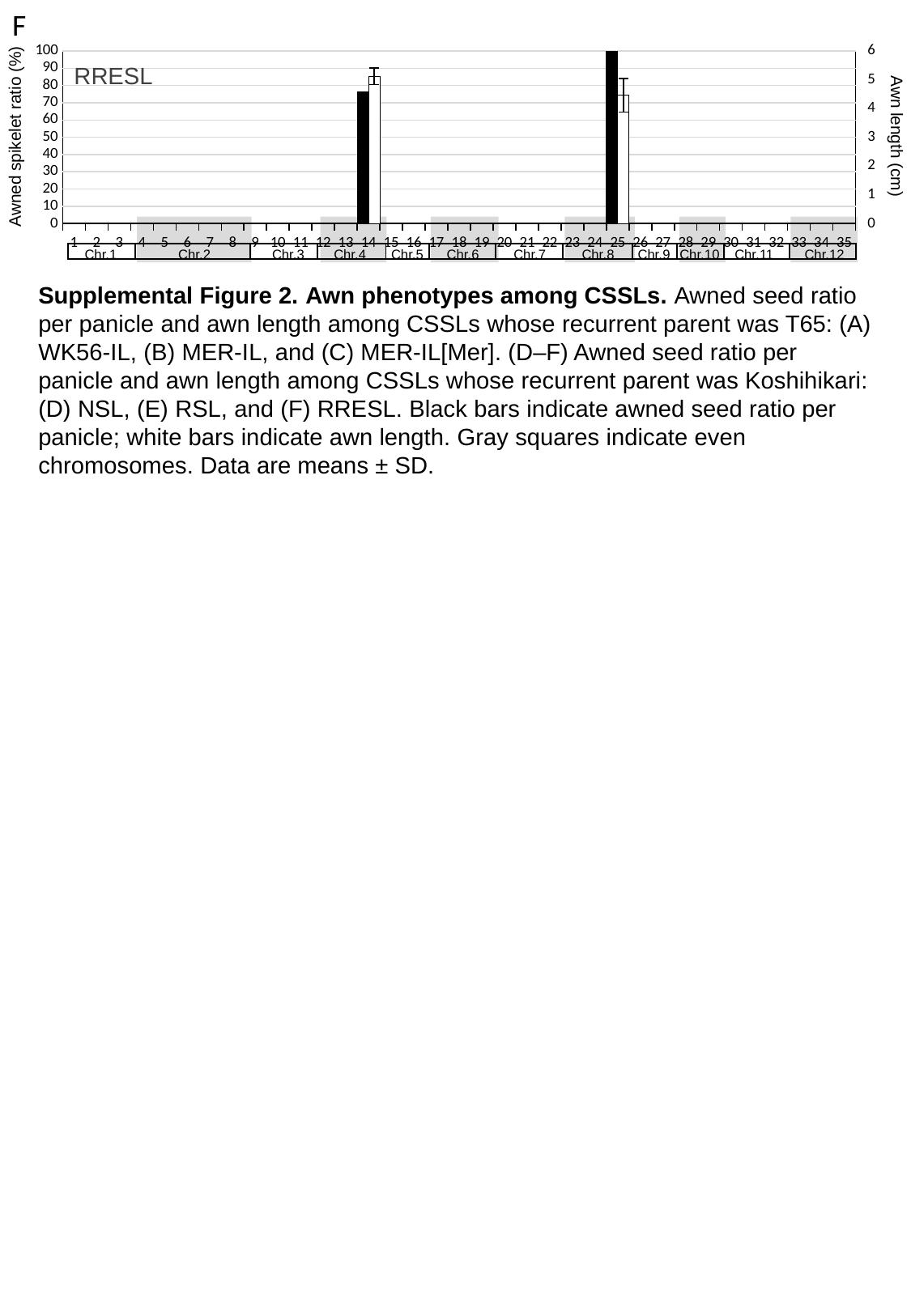

F
### Chart
| Category | awn ratio (%) | ダミー１ | ダミー２ | awn length(cm) |
|---|---|---|---|---|RRESL
Awned spikelet ratio (%)
Awn length (cm)
Chr.1
Chr.2
Chr.3
Chr.4
Chr.5
Chr.6
Chr.7
Chr.8
Chr.9
Chr.10
Chr.11
Chr.12
Supplemental Figure 2. Awn phenotypes among CSSLs. Awned seed ratio per panicle and awn length among CSSLs whose recurrent parent was T65: (A) WK56-IL, (B) MER-IL, and (C) MER-IL[Mer]. (D–F) Awned seed ratio per panicle and awn length among CSSLs whose recurrent parent was Koshihikari: (D) NSL, (E) RSL, and (F) RRESL. Black bars indicate awned seed ratio per panicle; white bars indicate awn length. Gray squares indicate even chromosomes. Data are means ± SD.

## Slide 4
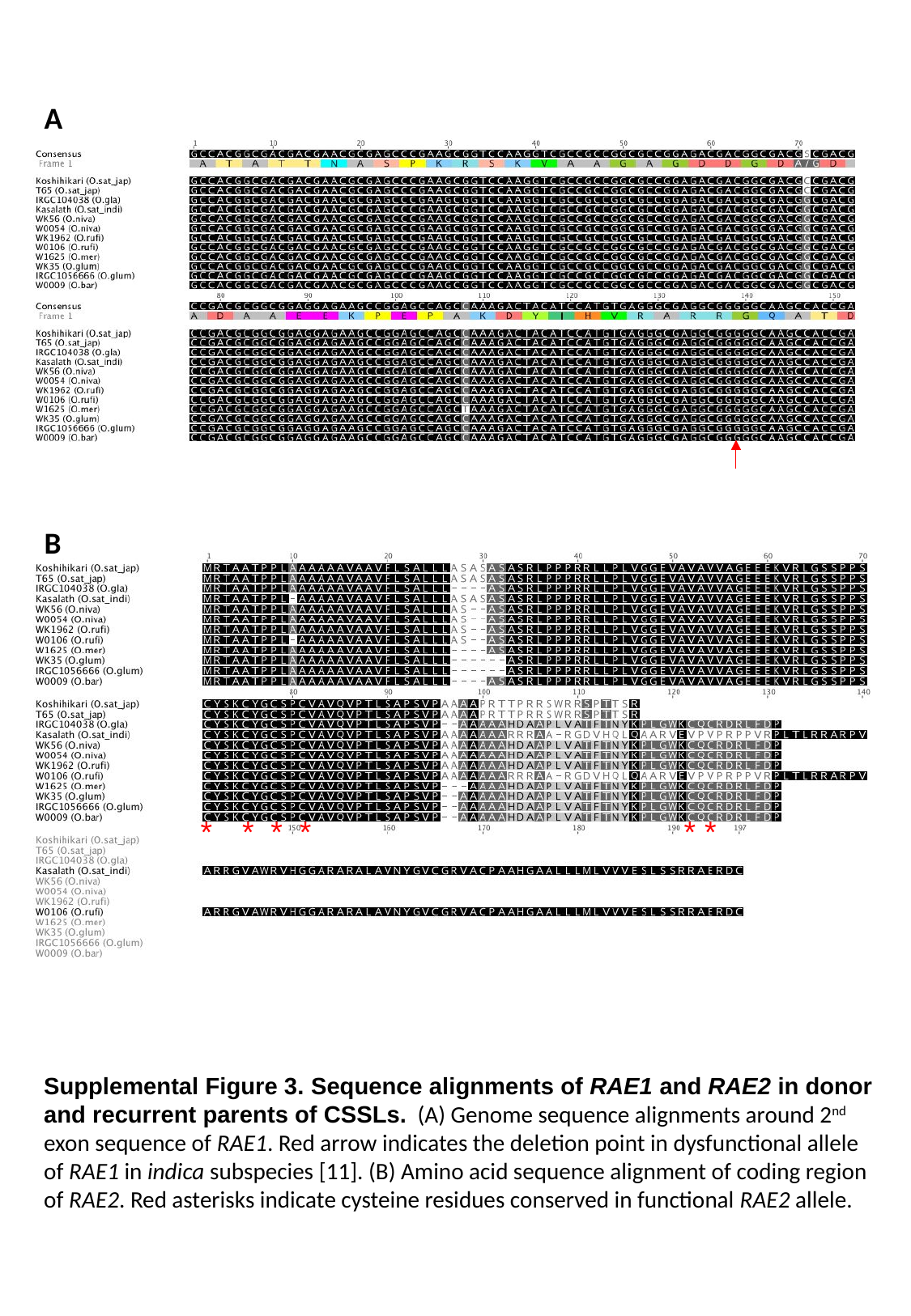

A
B
*
*
*
*
*
*
Supplemental Figure 3. Sequence alignments of RAE1 and RAE2 in donor and recurrent parents of CSSLs. (A) Genome sequence alignments around 2nd exon sequence of RAE1. Red arrow indicates the deletion point in dysfunctional allele of RAE1 in indica subspecies [11]. (B) Amino acid sequence alignment of coding region of RAE2. Red asterisks indicate cysteine residues conserved in functional RAE2 allele.

## Slide 5
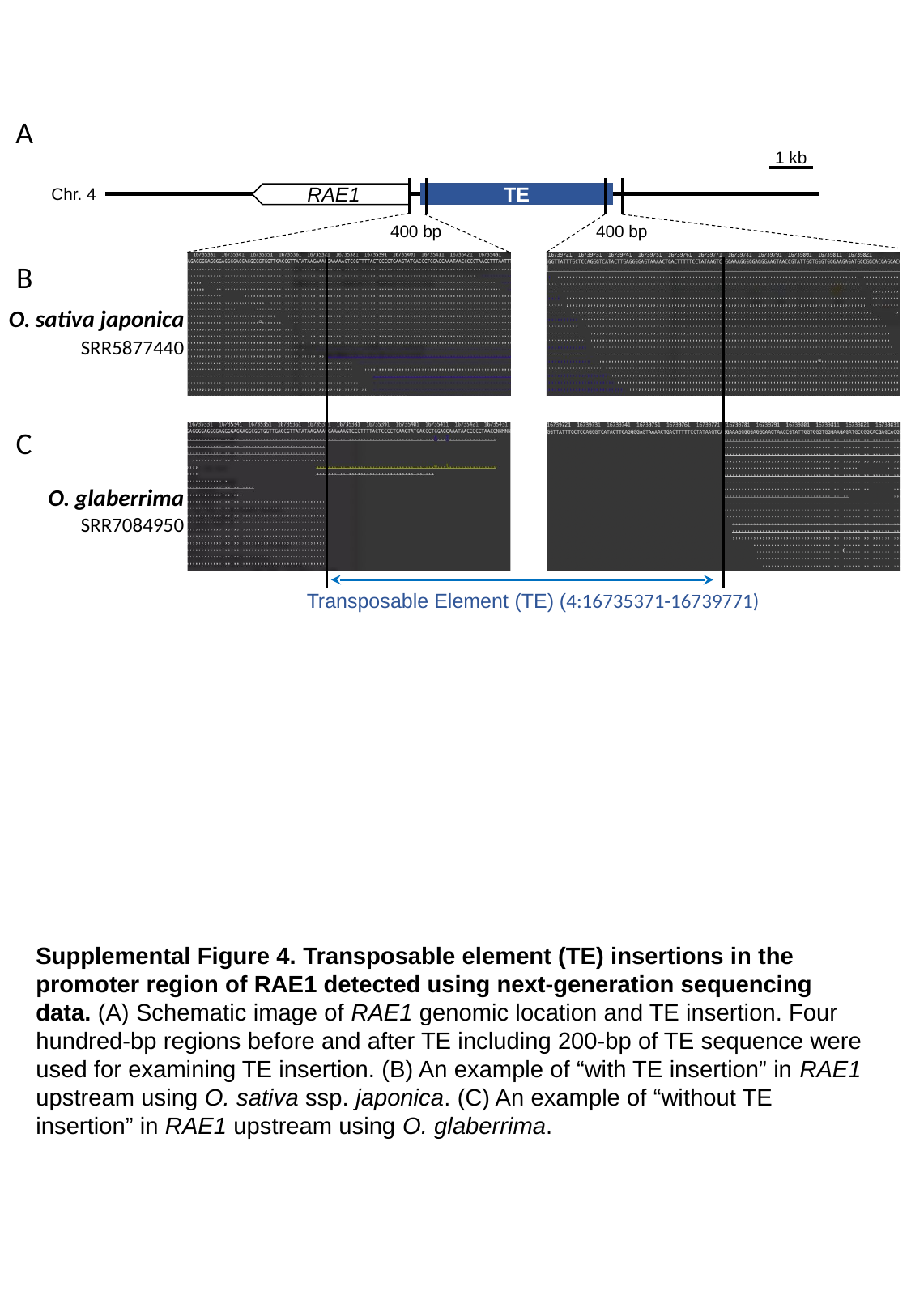

A
1 kb
TE
O. sativa japonica
SRR5877440
O. glaberrima
SRR7084950
Transposable Element (TE) (4:16735371-16739771)
Chr. 4
RAE1
400 bp
400 bp
B
C
Supplemental Figure 4. Transposable element (TE) insertions in the promoter region of RAE1 detected using next-generation sequencing data. (A) Schematic image of RAE1 genomic location and TE insertion. Four hundred-bp regions before and after TE including 200-bp of TE sequence were used for examining TE insertion. (B) An example of “with TE insertion” in RAE1 upstream using O. sativa ssp. japonica. (C) An example of “without TE insertion” in RAE1 upstream using O. glaberrima.

## Slide 6
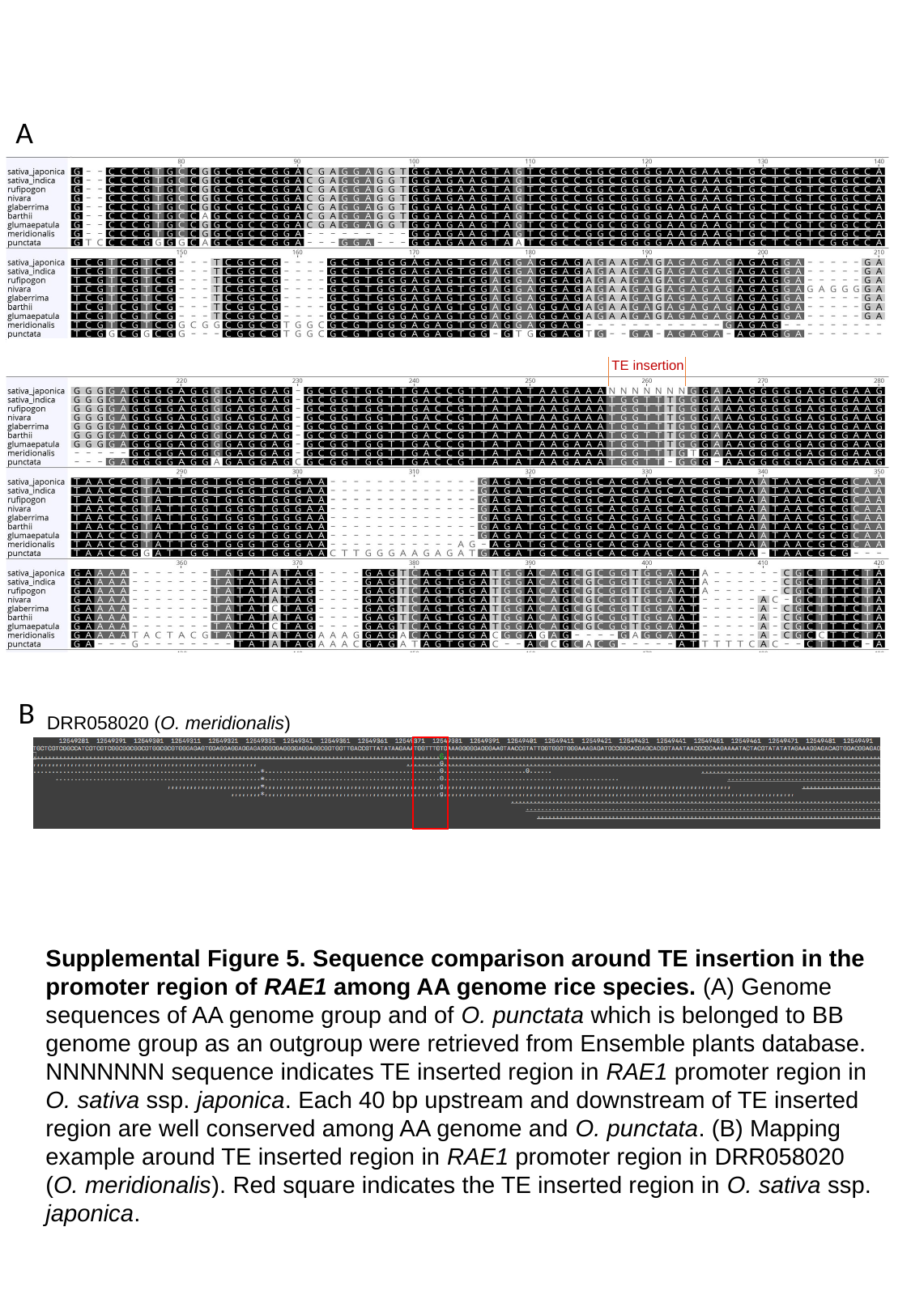

A
TE insertion
B
DRR058020 (O. meridionalis)
Supplemental Figure 5. Sequence comparison around TE insertion in the promoter region of RAE1 among AA genome rice species. (A) Genome sequences of AA genome group and of O. punctata which is belonged to BB genome group as an outgroup were retrieved from Ensemble plants database. NNNNNNN sequence indicates TE inserted region in RAE1 promoter region in O. sativa ssp. japonica. Each 40 bp upstream and downstream of TE inserted region are well conserved among AA genome and O. punctata. (B) Mapping example around TE inserted region in RAE1 promoter region in DRR058020 (O. meridionalis). Red square indicates the TE inserted region in O. sativa ssp. japonica.

## Slide 7
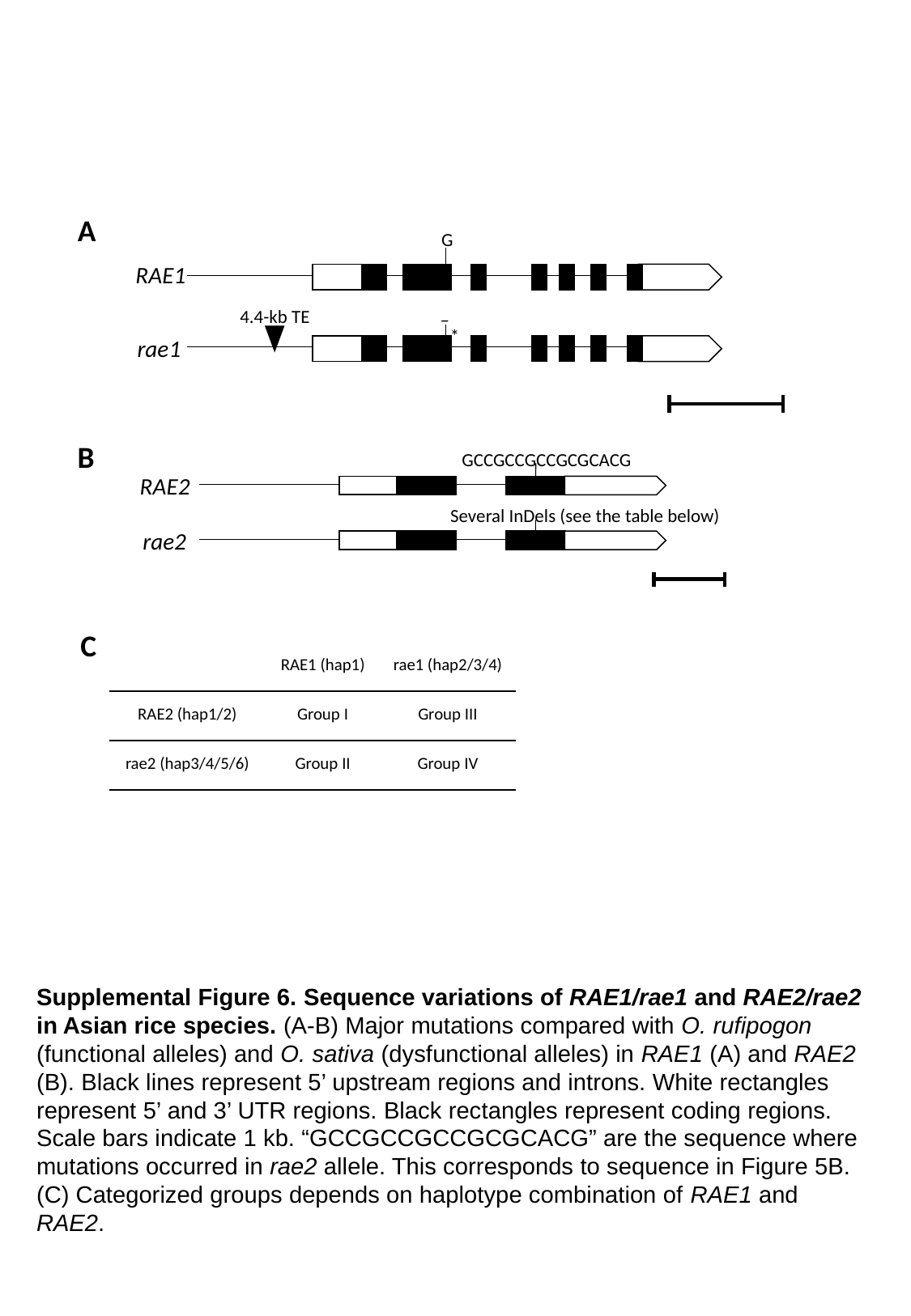

A
G
RAE1
4.4-kb TE
–
*
rae1
B
GCCGCCGCCGCGCACG
RAE2
Several InDels (see the table below)
rae2
C
| | RAE1 (hap1) | rae1 (hap2/3/4) |
| --- | --- | --- |
| RAE2 (hap1/2) | Group I | Group III |
| rae2 (hap3/4/5/6) | Group II | Group IV |
Supplemental Figure 6. Sequence variations of RAE1/rae1 and RAE2/rae2 in Asian rice species. (A-B) Major mutations compared with O. rufipogon (functional alleles) and O. sativa (dysfunctional alleles) in RAE1 (A) and RAE2 (B). Black lines represent 5’ upstream regions and introns. White rectangles represent 5’ and 3’ UTR regions. Black rectangles represent coding regions. Scale bars indicate 1 kb. “GCCGCCGCCGCGCACG” are the sequence where mutations occurred in rae2 allele. This corresponds to sequence in Figure 5B. (C) Categorized groups depends on haplotype combination of RAE1 and RAE2.
